# Updated applications of Ultrasound in Uterine Cervical Cancer

**DOI:** 10.7150/jca.49479

**Published:** 2021-02-22

**Authors:** Yi-Hsuan Hsiao, Shun-Fa Yang, Ya-Hui Chen, Tze-Ho Chen, Horng-Der Tsai, Ming-Chih Chou, Pang-Hsin Chou

**Affiliations:** 1 Institute of Medicine, Chung Shan Medical University, Taichung, Taiwan.; 2 School of Medicine, Chung Shan Medical University, Taichung, Taiwan.; 3 Department of Obstetrics and Gynecology, Changhua Christian Hospital, Changhua, Taiwan.; 4 Women's Health Research Laboratory, Changhua Christian Hospital, Changhua, Taiwan.; 5 College of Medicine, Kaohsiung Medical University, Kaohsiung, Taiwan.; 6 Department of Medical Research, Chung Shan Medical University Hospital, Taichung, Taiwan.

**Keywords:** cervical cancer, ultrasound, staging, therapy

## Abstract

Cervical cancer is a common gynecologic malignancy worldwide. It is the fourth for both incidence and mortality. For cervical cancer, imaging and pathology assessments are incorporated in the revised 2018 Federation of Gynecology and Obstetrics (FIGO) staging system. Uses of imaging techniques for the pre-treatment work-up of cervical cancer have been increasing. Among imaging techniques for the evaluation of cervical cancer, ultrasound is cheaper, faster and widely available than other imaging techniques such as computed tomography (CT) or magnetic resonance imaging (MRI). Advanced technique in ultrasound, such as three-dimension (3D) ultrasound and color Doppler, have improved the clinical application of ultrasound in cervical cancer. Ultrasound may provide highly accurate information on detecting tumor presence and evaluating local tumor extent if performed by ultrasound-trained gynecologists; the experience of readers is also critical for correct pretreatment staging and assessment of response to treatment. Sonographic images could be useful to predict response of neoadjuvant chemotherapy, radiotherapy, chemotherapy and concurrent chemoradiotherapy in patients with cervical cancer. This review article attempted to present the most updated specific applications of ultrasound in cervical cancer.

## Introduction

Cervical cancer is a common gynecologic malignancy among women. It is the fourth for both incidence and mortality in Global cancer statistics 2018 1. Nearly all cases of cervical cancer were resulted from human papillomavirus (HPV) infection 2. Most HPV infection is harmless but persistent infection with high-risk subtype of HPV (especially 16, 18) can cause cancer of the cervix 3. HPV vaccination program as the primary prevention and the Papanicolaou smear as cervical screening test as secondary prevention are effective strategies for the disease 4. Cervical cancer is often asymptomatic in the early stage. Clinical presentation may be post-coital or abnormal vaginal bleeding or a profuse malodorous vaginal discharge 4. Diagnosis is based on the histopathological assessment of a cervical biopsy. Squamous cell carcinoma (SCC) and adenocarcinoma (AC), the most common histological subtypes, are listed for approximately 70% and 25% of all cervix cancers, respectively 4.

Patient age, performance status, para-aortic lymph node status, tumor size, and pelvic lymph node status are significantly associated with progression-free interval 5. Clinical survey and pre-surgical tools to determine the extension of cervical cancer lesion for treatment approach and preventing the prognosis of the disease should be performed 5.

Cervical cancer can spread by lymphatic or hematogenous dissemination or direct extension. Staging cervical cancer is critical for approaching the most effective treatment strategy and predicting the outcomes. Stage IA cancer can be treated with cervical conization procedure or simple hysterectomy, while later stages may require neoadjuvant chemotherapy, radical surgery, radiation, chemotherapy or a combination of therapies. It is valuable to evaluate the response to treatment in cervical cancer women.

For cervical cancer, imaging and pathology assessments are incorporated in the revised 2018 Fédération internationale de gynécologie et d'obstétrique (FIGO) staging system 6. It is according to available resources for the use of the imaging modality to provide information on local or systemic spread, tumor size and nodal status 7. It was reported in a recent study that inclusion of imaging and surgical pathologic findings resulting in upward stage migration and mostly related to nodal and distant metastasis 8. Furthermore, incorporating the results of advanced imaging and surgical staging improved stratification of prognostic factors for relating survival outcomes comparing to the FIGO 2009 staging system for most cases by a cohort of 1282 patients 8. Different sonographic appearances of adenocarcinoma (AC) and squamous cell cancer (SCC) of the cervix was reported. The researchers demonstrated isoechoic or hyperechoic (relative to the surrounding stroma) in AC and hypoechoic in SCC using transvaginal ultrasound (TVUS) 9. This could be helpful in the clinical evaluation of cervical tumors.

Transvaginal and transrectal ultrasound (TVUS and TRUS) are typically performed by the gynecologist with the advantage of low cost and wide availability 6. The 2018 FIGO stages 3C1 and 3C2 (i.e. positive pelvic and/or paraaortic lymph nodes) can be assessed by the primary diagnostic work-up which includes diagnostic imaging to better assess tumor extent and metastatic disease 6.

A study enrolled 52 patients, 13 were advanced-stage disease (stage ≥IIb) and 39 were early-stage disease (stage ≤IIa), to evaluate the combined use of transvaginal elastography (TVES) and high-resolution TVUS in comparison with the diagnostic performance of magnetic resonance imaging (MRI) in detecting parametrial invasion in cervical cancer. It could be promising and economic for pre-operative work-up of cervical cancer by a dedicated gynecologic radiologist using TVES combined with TVUS 10.

In the cases with negative paraaortic lymph nodes on computed tomography (CT) or positron emission tomography (PET-CT), it could be necessary to perform dissection of the paraaortic lymph node in surgical staging. Obtaining an ultrasound or CT-guided tru-cut biopsy in equivocal extrauterine lesion is recommended to reduce false positive findings by imaging methods 11.

Hence, uses of imaging techniques for the pre-treatment work-up of cervical cancer have been increasing 12. Imaging modalities such as MRI, CT and positron emission tomography (PET) were used to develop treatment plans. However, these facilities are expensive, not universally available, extended scanning time required, intravenous contrast medium use and not real time. It is significant to deliberate upon an accurate, cost-effective and simple-to-use technology for measuring the tumor size, extent of stromal infiltration, and the invasion or parametrium, bladder or rectum.

**Figure 1:**
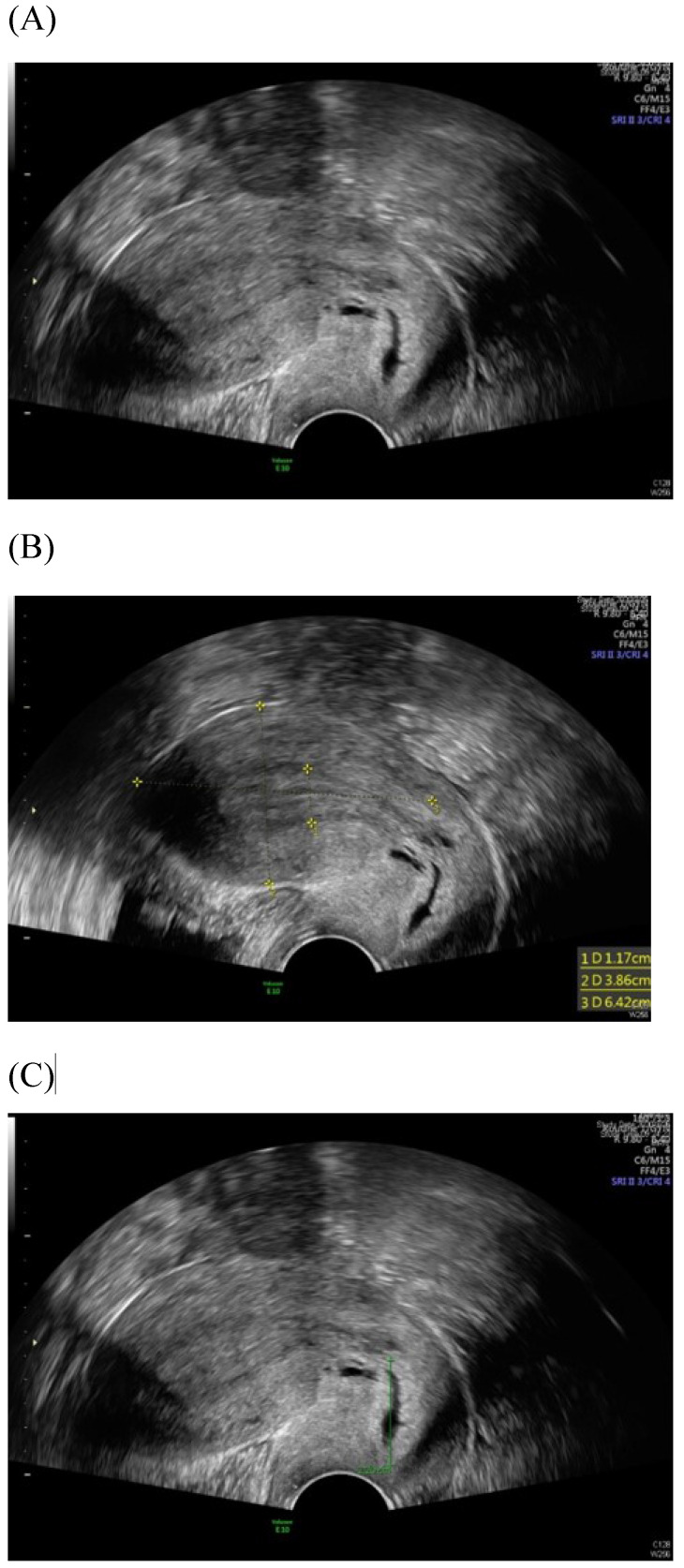
Transvaginal ultrasound image shows a longitudinal section of the uterus with normal cervix of the female aged 31-year-old. (A) Sagittal view (B) uterine size (C) cervical length.

**Figure 2:**
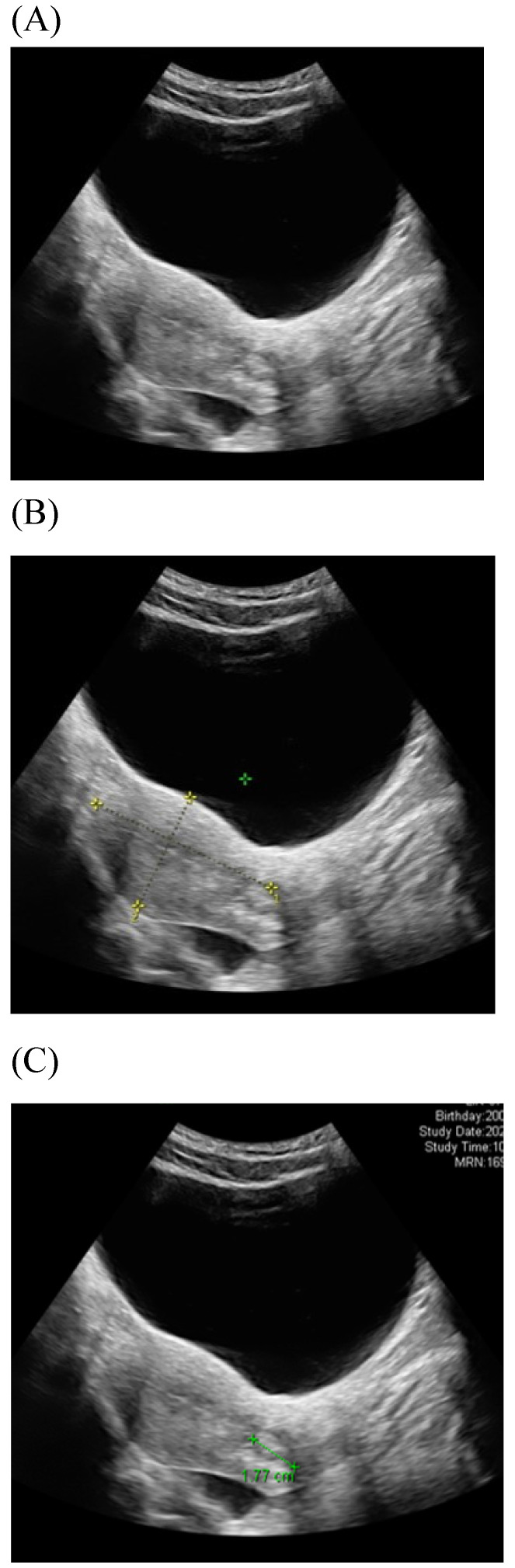
Transabdominal ultrasound image shows a longitudinal section of the uterus with normal cervix of the female aged17-year-old. (A) Sagittal view (B) uterine size (C) cervical length.

**Figure 3:**
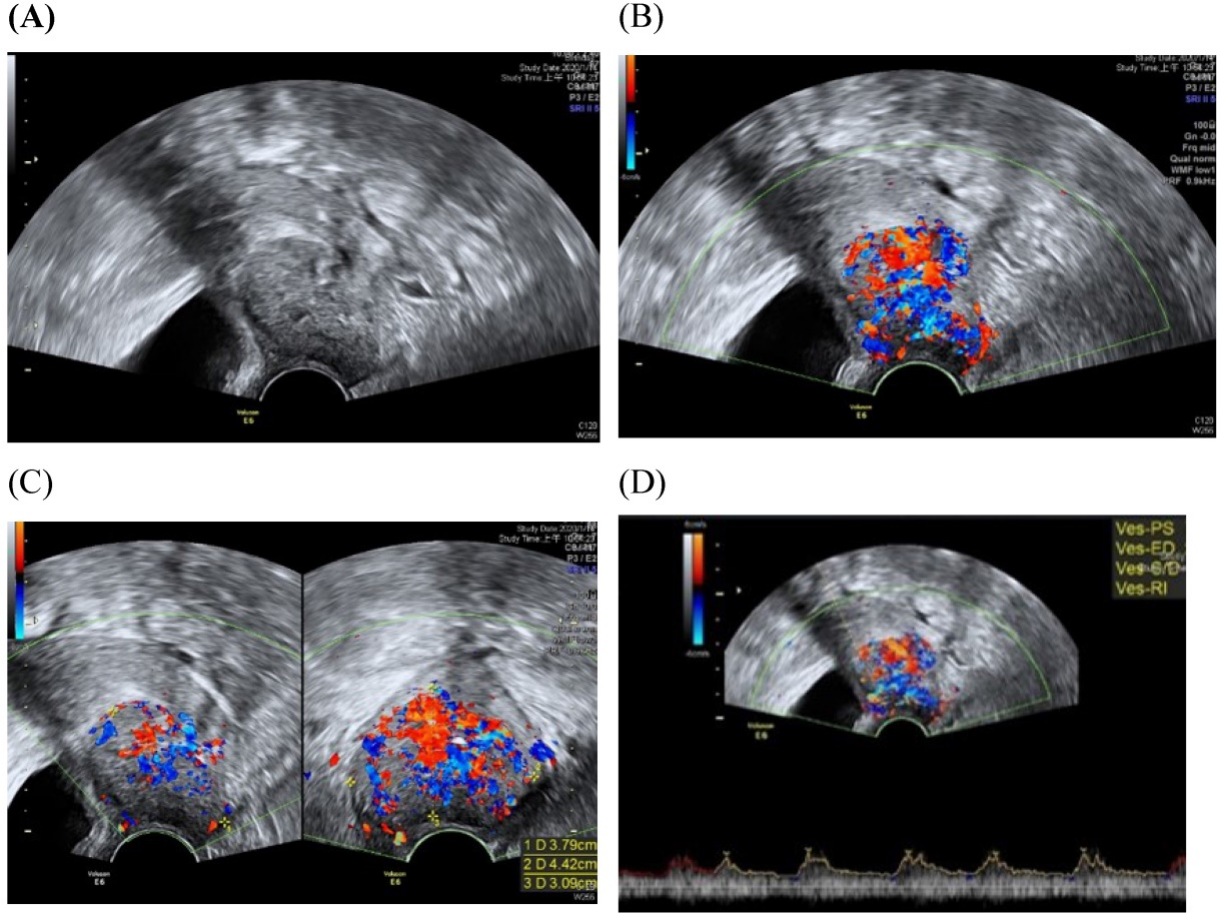
Transvaginall ultrasound image gray scale and color Doppler shows a longitudinal section of the uterus with cervical cancer of the female aged 68-year-old. (A) Gray scale shows cervical mass **(**B**)** color Doppler shows abundant blood flow in cervical mass (C) color Doppler (D) color Doppler Blood flow.

Among imaging techniques for the evaluation of cervical cancer, ultrasound is faster, radiation-free, cheaper, noninvasive, no contrast medium required and more widely available than other imaging techniques 13. Furthermore, advanced technologic developed in the field of ultrasound over the recent decades; image quality were improved. Transvaginal and transrectal high-frequency ultrasound, can give detailed images of the cervical tumor as the probe is positioned close to the tumor 13. Anatomy features of longitudinal sections of the uterus with normal cervix by (TVUS) examination and transabdominal sonography are shown in Figures 1 and 2. Furthermore, Figure 3 showed vascularity detected by transvaginal Color Doppler in cervical cancer. Ultrasound may provide highly accurate information on detecting tumor presence evaluating local tumor extent if performed by ultrasound-trained gynecologists 6. The experience of readers is also critical for correct pretreatment staging and assessment of response to treatment 6. This review article attempted to present the most updated specific applications of ultrasound in cervical cancer.

## Improving cervical cancer screening with ultrasound

The characteristics of the cervix blood flow in normal women and in women with precancerous lesions or invasive cancers were reported by using three-dimensional power Doppler ultrasound (3D PDU) indices, including the intensity of flow at the time of volume acquisition (FI), the number of vessels within the volume of interest (VI), and both blood flow and vascularization (VFI) 14. The study included 33 healthy controls, 71 cases of cervical cancer and 61 precancerous lesions. The three indices were all significantly higher in the cervical precancerous and cancer lesions groups than in controls (P < 0.001) 14. The index VI showed significantly higher in Stages IIIB-IV cancer patients than in those with less advanced disease (P = 0.045) 14. Improving cervical cancer screening with ultrasound could be significant, especial in women of reproductive age, in avoid overuse of cervical biopsy such LEEP or conization procedures. For improving cervical cancer screening with ultrasound, more studies, with a large number of participants, are necessary to establish reference values for 3D PDU indices in cervical lesions and application of the tool to assess the vascularity of cervical lesions.

TVCD combined with colposcopy was used to diagnose precancerous lesions and early stage cervical cancer 15. The study enrolled 100 patients, diagnosed precancerous lesions and early stage cervical cancer, were examined by TVCD and colposcopy 15. The study revealed the specificity, sensitivity and accuracy of combing TVCD with colposcopy significantly higher than that of TVCD (P < 0.05) 15.

## Local staging of cervical cancer

A thorough evaluation to determine the extent of cervical cancer should be performed if the diagnosis has been identified. There were several studies involving detection of the extension of cervical cancer lesion (Table 1).

The role of ultrasound for staging cervical cancer was reported in early 90s 16. Reliability of TRUS for staging of cervical cancer was reported previously 17. The study included 124 women with cervical cancer 17. The patients underwent examination with TRUS before treatment; sonographic findings were compared with the surgical pathologic stage.

The accuracies of staging with TRUS and clinical staging were 83% and 78%, respectively. The sensitivity, specificity and accuracy of TRUS for extent of parametrial involvement were 78%, 89% and 87%. TRUS could be helpful applied to routine pretreatment assessment of patients with cervical carcinoma 17. Tumor size is an important factor to predict the prognosis of patients with cervical cancer. The true volume of cervical carcinoma calculated more accurately by a 3D ultrasound system than 2D ultrasound was reported 18. Three-dimensional multiplanar sonography as a promising technique for the local staging of cervical cancer was demonstrated by the study designed of 14 patients with cervical carcinoma received transvaginal volume ultrasound examination prior to surgery. In 12 of the 14 cases, 3D ultrasound findings were compatible with pathology results 19. Ultrasound and MRI are highly accurate for the preoperative evaluation of women with early-stage cervical cancer 20. The study enrolled 209 women with early-stage cervical cancer (FIGO IA2-IIA) Complete data available for 182 patients 20. The diagnostic accuracy of ultrasound and MRI in the preoperative evaluation were performed 20. Ultrasound had better agreement with histology in assessing parametrial invasion (p<0.001) and residual tumor (p<0.001) compared than those results obtained by MRI 20.

In the assessment of parametrial infiltration in cervical cancer, a study included 29 patients using MRI and 2D and 3D ultrasound examination before treatment. The result showed that 2D and 3D ultrasound had similar moderate agreement with MRI. However, 2D and 3D ultrasound examinations are more available at low cost than MRI. Ultrasound could be considered as a method for preoperative work-up of cervical cancer 21.

The agreement of clinical examination, 3D and 2D sonography with MRI for local staging of cervical carcinoma was evaluated 22. Forty women were included and the results revealed 3D sonography showed good agreement with MRI for evaluating bladder involvement and parametrial infiltration in cervical cancer 22.

For evaluating the tumor's volume and intra-tumoral vascularization, the study enrolled 27 patients, with cervical cancer and stage 1B1 disease, who have an initial 2D ultrasound examination and followed by 3D volumes of the cervix. The Virtual Organ Computer Aided Analysis (VOCAL) program was used to calculate the tumor volume and vascularization 23. For tumors equal or smaller than 2.5 cm3 with linear vascularity, a significant superiority of 2D ultrasound over 3D VOCAL-PD was demonstrated; for larger tumor volume with complex vascularization, a superiority of 3D VOCAL-PD was revealed 23. Therefore, 3-D VOCAL-PD is superior to 2D ultrasound for calculating tumor volume and vascularization when tumor is more than 2.5 cm3 and show a complex vascularization in women with stage 1B1 cervical cancer 23.

A recent study reported the local staging of cervical cancer for 46 patients who underwent MRI of the pelvis and high‐resolution TVUS 24. The result showed a strong correlation between TVUS and MRI in the assessment of tumor volume in advanced stage and early stage cervical cancer. Ultrasound is a practical and economic imaging modality in local staging of cervical cancer 24.

A previous prospect study, included 38 cervical cancer patients, involved the TRUS in the evaluation of cervical carcinoma and comparing with spiral computed tomography (SCT) and MRI 25. In staging of early cervical cancer (less than stage 2b), the overall accuracy was 85% for examination under anesthesia (EUA), 65% for MRI, 75% for TRUS and 50% for SCT 25.

For locally advanced cervical cancer, 3-dimensional transvaginal ultrasound (3D-TVUS) was used to diagnose the extent of invasive cervical cancer and compare to that of Magnetic resonance imaging 26. For the detection of parametrial invasion: sensitivity was 75% with TVUS, 75% with MRI and 25% with clinical exanimation; specificity was 90% with TVUS and 55.6% with MRI; accuracy was 87.5% with TVUS and 59% with MRI. The authors claimed that preoperative 3D-TVUS may prove to be an excellent modality for the assessment of locally advanced cervical cancer 26.

For cervical primary tumor confined to the origin, the sonographic detection rate is high; transrectal or TVUS is a highly accurate modality in classifying early-stage tumors 27. It is important for the clinicians to plan the individual treatment of a patient 27.

The diagnostic performance of TVUS and MRI for assessment of presence and extent of cervical cancer was investigated by a prospective study 28. The patients with early stage cervical cancer planned for primary surgery and the patients with locally advanced cervical cancer arranged for surgery after neoadjuvant treatment. TVUS and MRI examinations presented the existence of the tumor in 56/60 (93%) and in 53/60 (88%) cases, respectively. Both ultrasound and MRI had similar sensitivity and specificity with reference to the parameters investigated such as the detection of the depth of stromal invasion to be greater than two-thirds. Ultrasound has the advantages over MRI of low priced and widespread availability 28.

The diagnostic performance of TVUS examination with regard to local extent and tumor size of the disease of early stage cervical cancer was investigated 29. Eighteen patients, with cervical carcinoma Stage IB1-IIA, were arranged for surgery with a TVUS examination. Histological examination showed parametrial infiltration in four patients; these were detected during ultrasound examination. TVUS examination is acceptably accurate modality for the assessment of tumor size and depth of cervical stroma invasion for clinical application 29.

Assessment of the extension of the lesion has been researched 30. A prospective multicenter study involved preoperative prediction of lymph node metastasis and deep stromal invasion in women with cervical cancer by using two-dimensional (2D) and 3D ultrasound indices 30. The results showed that 3D vascular indices were ineffective in speculating advanced stages of the disease 30. The previous study of TRUS imaging in staging of early cervical cancer revealed that TRUS imaging and the pathology-derived volumes correlated tightly 31. The high accuracy for detecting tumor (93.7%) was detected for TRUS, in detection of residual tumors following conization.

## Ultrasound findings as prognostic factors

Furthermore, angiogenesis, the formation of new vessels in a specific area, is required for tumor growth and progression 32. Table 2 showed studies involving ultrasound findings as prognostic factors of cervical cancer patients.

Transvaginal Color Doppler in cervical cancer allows non-invasive assessment of tumor angiogenesis 33. In Epstein et al study, Color Doppler signals were found in all cases of AC and 90% (18/20) of SCC cases; there was few detectable vascularization found in normal cervical tissue 9. Microvessel density correlated with survival for stage IB cervical cancer was assessed 34.

Color Doppler findings associated with risk factors was reported by a study involving neoangiogenesis measured in early cervical cancer cases 35.

Angiogenic parameters, including subjective assessment of pulsatility index (PI) and the amount of vessels within the tumor (scanty-moderate or abundant), were evaluated by transvaginal color Doppler ultrasound for 27 early stage cervical cancer patients. The risk factors (depth stromal invasion, parametrial and vaginal margin involvement, positive lymph nodes, lymph-vascular space involvement) were recorded. Tumors with abundant vasculization were significantly associated with parametrial involvement, lymph-vascular space involvement, pelvic lymph node metastases etc. Postoperative treatment was significantly more prevalent in patients with profuse vascularization 35.

The p53 protein expression in cervical cancer after RT correlated with transvaginal color Doppler ultrasound findings was reported by a recent study 36. In the study, RT was performed to the 78 enrolled patients. Morphological features of tumor tissues after RT and the mutant p53 protein level were detected via immunohistochemistry (IHC). Blood flow signals measured by resistance index (RI) by transvaginal color Doppler ultrasound. The p53 level was significantly correlated with color Doppler flow imaging grading and RI (p<0.05). The level of p53 and transvaginal color Doppler ultrasound could provide valuable clinical information for the treatment and monitoring of cervical cancer 36.

## Prediction of treatment response

Several studies have evaluated the role of ultrasound for predicting the response to varied treatment in women with cervical cancer.

### Prediction of response to neoadjuvant chemotherapy

The role of TVUS in early prediction of pathological response in a large series of locally advanced cervical cancer patients arranged neoadjuvant treatment followed by radical surgery was investigated by using 3D PDU 37. The result showed that Low vascularization index (VI) predicts poor treatment response in LACC 37. Tumor volume, 3D power Doppler indices were obtained before (baseline survey) and after 2 weeks of treatment for 108 women with LACC Stage IB2-IVA. Among the 108 patients, 88 were included in the final analysis. After 2 weeks of neoadjuvant treatment, patients with partial response had significantly greater tumor volume than those with complete response (P = 0.019). Before and after 2 weeks of treatment, the partial response showed significantly lower vascularization index (VI) than the complete-response group 37.

Using Power Doppler vascularity index (PDVI) to predict the response to neoadjuvant chemotherapy (NACT) in bulky early stage cervical carcinoma was reported 38. For twenty-five women with bulky early stage cervical cancer treated by NACT followed by surgery, transvaginal power Doppler was performed to evaluate their response to NACT 38. Twelve (48%) patients showed a response to NACT and thirteen (52%) were unchanged or had progressive disease after NACT. Tumors with pelvic lymph node metastasis and lymphovascular emboli showed Higher PDVI values both before and after NACT 38. The power Doppler vascularity index could be useful to predict the response to neoadjuvant chemotherapy in cervical cancer 38.

A prospective study involved 42 women with locally advanced cervical cancer referred for NACT 39. TRUS and MRI used to evaluate tumor size following neoadjuvant chemotherapy for women with locally advanced cervical cancer was conducted 39. TRUS could be an accurate diagnostic modality in the evaluation of tumor volume after neoadjuvant chemotherapy (NACT) in patients with cervical cancer 39.

Transvaginal Color Doppler sonography used to predict the response to chemotherapy in patients with advanced cervical cancer was reported 40. Twenty-three patients with cervix cancer (FIGO stages Ib bulky-IIIb) and twenty healthy women received transvaginal color Doppler assessment of the intracervical vessels and the uterine arteries. A decrease in peak systolic velocity and a significant increase in resistance and pulsatility index values were noted in ten patients who responded to chemotherapy. Doppler parameters could be clinically useful in the assessment of the response to neoadjuvant chemotherapy 40. In a recent study, the researchers showed that 2D/3D ultrasound is useful to evaluate the response to neoadjuvant chemotherapy (NACT) in LACC 41.

### Prediction of response to neoadjuvant chemoradiation

The residual tumor in locally advanced cervical cancer patients receiving chemoradiation and radical surgery was assessed by 3D power Doppler, 2D ultrasound parameters, or contrast-enhanced indices 42. The results showed that grayscale and color Doppler ultrasound have low levels of diagnostic performances of detecting residual disease after neoadjuvant chemoradiation for locally advanced cervical cancer patients 42.

Ttransvaginal color Doppler ultrasonography (TCD) used to predict response to preoperative chemoradiation for locally advanced cervical carcinoma patients was investigated 43. The research included ten patients with locally advanced cervical cancer who were arranged for preoperative chemoradiation and underwent TCD examination prior to beginning the treatment protocol. The parameters were calculated, including the ratio between the number of vessels, intra-tumor vessels and tumor volume and resistance index (RI). Those tumors with Complete pathological response (pathCR) had lower TVD (0.1 vs. 1.1, p = 0.05), lower mean number of vessels (3.3 vs. 5.3, p = 0.01), and higher RI (0.41 vs. 0.29, p = 0.03) 43. For patients with locally advanced cervical cancer, TCD could be used to predict response to preoperative chemoradiation 43.

### Prediction of response to radiotherapy

Alterations in cervical intra-tumoral vascularization during and after radiotherapy investigated by 3D PDU was reported 44. 3D-PDU detected local disease with 98.5% specificity and 75.0% sensitivity at follow-up. Among 34 patients with squamous cell carcinoma, Serum markers detected local disease with 20.0% sensitivity and 77.3% specificity. In comparison of serum markers in cervical squamous cell carcinoma, 3D-PDU showed higher sensitivity and specificity in detecting local recurrence or persistence in cervical cancer 44. The authors suggested that combination of 3D-PDU and clinical assessment could be a new and safe method for detecting local recurrence and monitoring radiotherapy treatment response 44.

The evaluation of radiotherapy response of cervical cancer by suing gray scale and color Doppler ultrasonography was report 45. This study included 13 patients with advanced stage cervical cancer underwent MRI and transvaginal color Doppler ultrasonography examinations 6 months prior to and 6 months after radiotherapy. There was a significant difference between increased resistive indices and complete response to the treatment (P=0.001). A significant correlation existed between the MRI findings and resistive indices. The parameters obtained with TVCDUS could be a good alternative to MRI to evaluate the response of cervical cancer to the treatment 45.

### Response to concurrent chemoradiation

It was reported that Transvaginal color Doppler sonography could be useful in predicting clinical response to concurrent chemoradiation for locally advanced cervical carcinoma 46. There were 21 patients with locally advanced cervical cancer included. The patients underwent the assessment of Tumor vascularity by TVCD before the start of concurrent chemoradiotherapy. The parameters included the lowest pulsatility index (PI), lowest resistance index (RI), and highest peak systolic velocity (PSV) from central vessels intra-tumor 46. Complete clinical response (CR) was achieved in 11 patients (52%), and 10 (48%) had partial clinical response (PR). PI was higher in tumors presenting CR than in those with PR (p < 0.01); RI was higher in those tumors presenting CR than in those with PR (p < 0.01). No differences were found in PSV 46.

The investigation of early response of locally advanced cervical cancer patients undergoing concurrent chemo-radiotherapy (CCRT) by ultrasound was reported 47. B-mode ultrasound examinations were performed for thirty-four patients during CCRT. The echogenicity, homogeneity and heterogeneity of tumors were calculated in reference of six ultrasonic histogram parameters 47. The peak intensity value of the histogram increased rapidly since the first week after therapy initiation; the width of the low-intensity and the high-intensity and the area under high intensity portions of the curve changed significantly at the second week. It was concluded that ultrasonic histogram could be a potential marker in monitoring early response during CCRT 47.

## Application of ultrasound in intraoperative guidance

The role of intraoperative ultrasound guidance in intracavitary brachytherapy of cervical cancer was investigated in a recent retrospectively study 48. Ultrasound guidance was performed for tandem selection and appropriate application. The applicator conformity were evaluated with planning CT. Intracavitary brachytherapy was done under ultrasound guidance for 412 insertions in 113 patients with cervical cancer. With ultrasound guidance, one of 113 patients suffered uterine perforation (0.9%), tandem was in myometrium in one patient (0.9%) and tandem length was short in one patient (0.9%). The authors concluded that intraoperative sonographic guidance provided effectiveness for intracavitary brachytherapy of cervical cancer and decreased rates of perforations 48. The previous study, involving intraoperative ultrasound guidance while intracavitary brachytherapy applicator placement for cervical cancer patients, also showed the same results 49. Intraoperative ultrasound guidance increased the rate of successful applicator placement 49.

In a recent systematic review and meta-analysis, a decrease of uterine perforations by using ultrasound image-guided applicator insertion for cervical carcinoma patients underwent intracavitary brachytherapy was revealed 50.

TRUS guided high dose rate (HDR) interstitial brachytherapy for patients of cervical cancer was investigated 51. This study included 25 cervical cancer patients, suitable for intracavitary radiotherapy (ICRT), who were given two weekly sessions of HDR IBT each after pelvic external beam radiation therapy. The investigators claimed that it was helpful in accurate placements of needles resulted in avoiding the injury to normal pelvic structures 51.

## Conclusion

For the ability of evaluating morphology and intensity of blood flow, ultrasound technique might be used to improve cervical cancer screening, and this need more studies to confirm the applications. Imaging during the primary diagnostic work-up is essential for accurately assessment of tumor extent and selecting the best therapeutic option for cervical cancer patients. Ultrasound may be a useful modality to evaluate local extent of disease in cervical cancer. This technique is limited for evaluating lymph node status. Tumor vascularization by Doppler ultrasound could be useful for monitoring and predict response to therapy.

## Figures and Tables

**Table 1:** Application of ultrasound in evaluation of uterine cervical cancer and extension of the disease

Ultrasound Imaging modality	Number of patients evaluated	Performance	Reference
Transrectal US	124	In pretreatment evaluation of patients with cervical carcinoma by TRUS, the accuracy of staging with TRUS was 83%.	Innocenti P et al. 17
Transvaginal 3D and 2D sonographic scans	61	In comparing volume estimation of cervical carcinoma from specimen and tumor volume determined by 3D or 2D ultrasound, the results showed +6.68 to -6.10 mL for 3D assessment and +12.46 to -10.98 mL for 2D estimation.	Chou CY et al. 18
High-end US systems either transvaginal or transrectal	182	For parameters assessed on US / MRI and compared to pathology, US showed better assessing in residual tumor (p<0.001) and parametrial invasion (p<0.001).	Epstein E et al. 20
2D and 3D transvaginal US	29	To assess parametrial infiltration in cervical cancer, the high consistency, between 3D US and MRI, was detected in the middle cervical cylinder (76%; kappa, 0.438)	Chiappa V et al. 21
2D and 3D Transvaginal Sonography	40 (11 early-stage and 29 advanced-stage disease)	Agreement for parametrial infiltration between 3D US and MRI (κ = 0.60; 95% CI, 0.35-0.85; 80.0% agreement); Agreement for bladder involvement between 3D US and MRI (κ = 0.84; 95% CI, 0.55-1.0; 97.5% agreement)	Arribas S et al. 22
3D vocal power Doppler US VS 2D US	27	In assessing tumor volume and intra-tumoral vascularization, 3D VOCAL-PD showed superior in detecting larger volume tumors with complex vascularization than 2D US; 2D US showed better performance in assessing tumors equal or smaller than 2.5 cm^3^ with linear vascularity.	Daskalakis G et al. 23
High-resolution transvaginal US	46	In detecting parametrial invasion, sensitivity rates 86% for transvaginal US.	Moloney F, et al. 24
3D transvaginal US	24	For cancer staging, accuracy was 66.7% with transvaginal US. For the detection of parametrial invasion, sensitivity was75% with transvaginal US.	Byun JM et al. 26
Transrectal US	95	The accuracy of tumor detection by transrectal US was 90.5% in detecting small tumors (≤1 cm (3)); The accuracy of parametrial infiltration recognized by Transrectal US was 98.9%.	Fischerova D et al. 31

FIGO, the International Federation of Gynecology and Obstetrics; 3D, three-dimensional; 2D, two-dimensional; US, ultrasound; MRI, magnetic resonance imaging.

**Table 2:** Ultrasound findings as prognostic factors of cervical cancer patients

Ultrasound Imaging modality	Number of patients evaluated	Performance	Reference
Transvaginal color Doppler ultrasound	27 patients early stage invasive cervical cancer	“Abundant” vascularization and pulsatility index (PI) < 0.82 could be related to postoperative treatment because of risk factors.	Jurado M et al. 35
Transvaginal color Doppler ultrasound	78 patients with cervical cancer (stage II and III)	The p53 protein expression level was significantly correlated with color Doppler flow imaging grading and blood flow resistance index (RI) (p<0.05).	Wang P et al. 36
